# A Case of Mixed Infections in a Patient Presenting with Acute Febrile Illness in the Tropics

**DOI:** 10.1155/2013/562175

**Published:** 2013-02-27

**Authors:** L. S. Yong, K. C. Koh

**Affiliations:** ^1^Department of Medicine, Hospital Tuanku Ja'afar Seremban, Jalan Rasah, 70300 Seremban, Negeri Sembilan, Malaysia; ^2^Department of Medicine, Clinical School, International Medical University, Jalan Rasah, 70300 Seremban, Negeri Sembilan, Malaysia

## Abstract

Concurrent infections with more than one etiological agent can result in an illness with overlapping symptoms, resulting in a situation where the diagnosis and management of such a patient could be challenging. We report a case of *vivax* malaria in a patient who was also serologically positive for leptospirosis and dengue.

## 1. Introduction

Mixed infections are not uncommon in the tropics. Dengue usually presents with symptoms of flu-like illness such as high-grade fever, generalized body ache, arthralgia, myalgia, nausea, and vomiting as well as maculopapular rashes. The symptoms of dengue may mimic other diseases such as leptospirosis and malaria which are also prevalent in areas where dengue is endemic [1]. The similarity in symptomatology between these 3 infectious diseases may sometimes complicate the diagnosis of a patient presenting with acute fever. Laboratory investigations are often necessary to arrive at a definite diagnosis. However, interpreting laboratory results in the setting of mixed infection may prove to be challenging. For instance, a patient who presents with acute febrile illness in the tropics and is found to be dengue IgM serology positive should not be automatically assumed to have dengue infection. The astute clinician should look for other causes of fever if atypical presentations in the patient arouse suspicion of the presence of other possible etiologies.

We report a case of a patient who presented with fever and thrombocytopenia with multiple positive laboratory results pointing to several possible etiologies. We would like to highlight several learning issues from this case.

## 2. Case Report

A 47-year-old man from Pakistan initially presented with complaints of high grade fever with rigors of 6-day duration which was associated with throbbing headache, periorbital pain, generalized body aches, myalgia, fatigue, and anorexia. He denied any bleeding tendencies or skin rash.

The patient worked as a waiter in a local restaurant. There was no significant past medical history or travel history. Although he lived in an urban area known to be endemic for dengue, no recent fogging has been carried out in that area. 

On examination, the patient was conscious and oriented but was mildly dehydrated. No skin rashes or hemorrhagic manifestations were observed. His blood pressure was 100/70 mmHg and the pulse rate was 100/min which was regular and of good volume. His temperature was 38.3°C. Examinations of the respiratory, cardiovascular, and gastrointestinal systems were unremarkable.

An initial diagnosis of dengue in the febrile phase of the illness was made based on the presenting features of this patient and a positive dengue IgM result using IgM capture enzyme-linked immunosorbent assay (ELISA). The patient also had thrombocytopenia which was consistent with the diagnosis of dengue (Table 1). He was managed with appropriate supportive therapy and hydration with crystalloid fluids according to established guidelines for dengue [2]. Despite this, the patient remained febrile on day 8 of illness. 

At this point, the differential diagnoses of malaria and leptospirosis were considered. The leptospirosis IgM dipstick test was reported to be positive. The patient was then commenced on a daily dose of 1 g Ceftriaxone, which was administered intravenously for 7 days. On the same day, a peripheral blood smear collected during a temperature spike was reported to be positive for the presence of *Plasmodium vivax* with 3,800/uL of asexual forms (Table 1). A dosage of oral Chloroquine at 600 mg was initiated immediately followed by another dose of oral Chloroquine 300 mg at 6 hours after the first dose and 300 mg daily for the next two days as per established guidelines for the treatment of chloroquine sensitive *vivax* malaria [2]. The patient was also started on daily oral Primaquine 30 mg for 14 days to eradicate hypnozoites in the liver. The blood cultures did not yield any positive growth while the microscopic agglutination test (MAT) for leptospirosis sent on day 8 of illness was reported to be positive with titer of 1 in 400. The MAT was repeated 25 days after the first MAT sample was still positive with a lower titer of 1 in 100.

After the commencement of antibiotics and antimalarial agents, the patient's condition improved remarkably with defervescence within 24 hours followed by normalization of platelet count over the next 48 hours. Throughout the illness, the renal and liver function tests of the patient were within normal reference range. By the eighth day of hospitalization, the blood film for malaria parasite was clear.

## 3. Discussion

Our patient initially presented with symptoms of a nonspecific viral-like illness. The diagnosis of dengue should be considered in a patient presenting with acute febrile illness if he originated from a dengue endemic area. Dengue infection is caused by dengue virus which is a mosquito-borne flavivirus. It is transmitted by the bites of infected female mosquitoes, *Aedes aegypti* and *A. albopictus*. The incidence of dengue in Malaysia is estimated to be 181 per 100,000 population [3]. It is a notifiable disease in Malaysia and following the notification, the public health department would carry out campaigns to search for and destroy *Aedes *breeding sites including newly recognized potential breeding sites such as roof gutters, usage of larvicide in domestic water containers, and preventive insecticide fogging in the area where a case has been reported [4].

 Dengue virus infection causes a spectrum of clinical manifestation ranging from mild illness to fatal hemorrhagic disease. However, in this patient, several features of his illness were inconsistent with the diagnosis of dengue such as the protracted febrile phase lasting beyond a week and the absence of leucopenia and hemoconcentration in the laboratory investigation results (Table 1). Although the dengue IgM serology was positive, it does not necessary indicate a recent exposure to dengue virus infection, as the dengue IgM level can remain elevated for up to several months after exposure. In addition, the test has been reported to cross-react with nonflavivirus infections such as malaria, leptospirosis, toxoplasmosis, and syphilis [5]. In our centre, the Panbio Dengue IgM Capture ELISA test (MediMark Europe, France) was used for the detection of dengue IgM and this test has been reported in the product leaflet of the test kit to have a 10% cross-reactivity with malaria but no cross-reactivity with leptospirosis.

Leptospirosis is a zoonotic infectious disease caused by pathogenic spirochete bacteria of the genus *leptospira* that is transmitted directly or indirectly from infected animals (usually rodents) to human. The etiological agent is *Leptospira interrogans*, which has more than 200 serovars with 25 serogroups [6]. Leptospirosis has a broad range of clinical manifestations, ranging from mild flu-like illness to very severe disease with hemorrhagic manifestations and multiorgan failure. In the absence of definitive characteristic clinical features, especially in mild leptospirosis, diagnostic laboratory investigations are often helpful.

In this patient, although there were no obvious risk factors and clinical features suggestive of leptospirosis, the protracted illness with thrombocytopenia in a tropical country such as Malaysia should prompt the clinician to consider its diagnosis. The diagnosis of leptospirosis in this patient was made based on the microscopic agglutination test (MAT) for leptospira antigen titer of ≥1 : 400 [7]. 

Leptospirosis is sometimes referred to as “The Great Mimicker” and it may be overlooked and underdiagnosed due to its varied clinical presentations. In the early stages of the illness, leptospirosis is often indistinguishable from other common causes of acute febrile illnesses in the tropics such as dengue, malaria, and typhoid [8, 9]. In a hospital-based cross-sectional study conducted among 10 healthcare facilities in Northeastern Malaysia, only 31% of MAT confirmed leptospirosis cases were diagnosed as leptospirosis on discharge from the hospital while another 38% were erroneously diagnosed as dengue fever or dengue hemorrhagic fever [10].

Likewise, leptospirosis and malaria share many similar symptoms and signs such as headache, acute fever, myalgia, malaise, lethargy, rash, pallor, jaundice, bleeding tendencies, and hepatosplenomegaly. Due to the overlapping symptoms and signs, malaria may be overlooked in the presence of positive leptospirosis IgM. As malaria and leptospirosis are common in the tropics, coinfections with these two pathogens are not uncommon although they have rarely been reported. Two cases of malaria-leptospirosis coinfections have been reported from Thailand near the Thailand-Myanmar border. The first case was a patient with *Plasmodium falciparum *infection who also had acute and convalescent MAT titers for *Leptospira serovar icterohaemorrhagiae *of 1 : 200 and 1 : 1,600, respectively. The second case was of a patient with *P. vivax *infection associated with markedly elevated MAT titer of 1 : 3,200 for *Leptospira serovar bataviae *[11]. On the other hand, there has been no report of *vivax* malaria associated with false positive MAT. 

Although there were no obvious predisposing factors noted from the patient's history, we had strong reasons to believe that this patient was coinfected with malaria and leptospirosis. The evidences include the presence of *Plasmodium vivax* in the peripheral blood, the positive leptospira IgM serology, the significantly elevated MAT titer, and the patient's remarkable recovery after initiation of antimalarial and appropriate antileptospirosis therapies. We believe that the positive dengue IgM serology detected early in the patient's illness was most likely due to cross-reactivity of the laboratory test with malaria parasites, which proved to be a red-herring in masking the real diagnosis in this patient. The repeat MAT for leptospirosis after 25 days showed a reduction in titer from 1 : 400 to 1 : 100, indicating successful treatment of the leptospirosis.

## 4. Conclusion

Coinfections with several pathogens such as dengue, malaria, and leptospirosis are not uncommon in areas of high endemicity for these pathogens in patients presenting with acute febrile illness with atypical manifestations. Interpreting the multiple positive serological results in this setting may be challenging. However, meticulous history taking, careful clinical examination, and careful interpretation of laboratory results based on available evidence will often lead to the correct diagnosis. 

There is a need for greater awareness amongst clinicians of the possibility of coinfections in a patient presenting with acute febrile illness with subtle atypical manifestations. The optimal use of microbiological laboratory services aiding the diagnostic process is also required. The diagnosis in our patient could have benefited from more definitive and rapid diagnostic tests such as the polymerase-chain reaction (PCR) if available.

## Figures and Tables

**Table 1:** Laboratory results on the first, third, and eighth day of hospitalization, respectively.

Laboratory result	Reference range	Day of illness (day of hospitalization)*		
		6 (1)	8 (3)	13 (8)
Hemoglobin	13.0–17.0 g/dL	13.8	13.4	13.7
Total white cells count	4.0–11.0 × 10^9^/L	6.4	5.5	8.3
Platelet count	150–400 × 10^9^/L	67	80	179
Hematocrit	40–50%	40.9	39.2	
Serum creatinine	74–110 umol/L	101	102	
Blood urea	2.8–7.2 mmol/L	2.3	4.2	
Serum alanine transaminase	<45 U/L	55	40	
Dengue IgM (ELISA)		Positive		
Leptospira IgM rapid test			Positive	
Leptospira titer (MAT)			1 : 400	Clear
Blood film for malaria parasite			*P. vivax* detected.	

*Days 1, 3, and 8 of hospitalization corresponded with days 6, 8, and 13 since onset of fever, respectively.

MAT: microscopic agglutination test; *P. vivax*: *Plasmodium vivax*.
